# Research progress on distribution and exposure risk of microbial aerosols in animal houses

**DOI:** 10.3389/fvets.2022.1015238

**Published:** 2022-11-10

**Authors:** Cheng Lou, Yu Bai, Tongjie Chai, Hui Yu, Tuorong Lin, Guangming Hu, Yuling Guan, Bo Wu

**Affiliations:** ^1^Guangdong Provincial Key Laboratory of Animal Molecular Design and Precise Breeding, School of Life Science and Engineering, Foshan University, Foshan, China; ^2^College of Veterinary Medicine, Shandong Agricultural University, Tai'an, China; ^3^Key Laboratory of Animal Bioengineering and Animal Disease of Shandong Province, Tai'an, China; ^4^Sino-German Cooperative Research Centre for Zoonosis of Animal Origin Shandong Province, Tai'an, China

**Keywords:** farm animal, microbial aerosol, distribution characteristics, exposure risk, transmission

## Abstract

Environmental aerosols in animal houses are closely related to the productive performance and health level of animals living in the houses. Preferable housing environments can improve animal welfare and production efficiency, so it is necessary to monitor and study these environments. In recent years, there have been many large-scale outbreaks of respiratory diseases related to biological aerosols, especially the novel coronavirus that has been sweeping the world. This has attracted much attention to the mode of aerosol transmission. With the rapid development of large-scale and intensive breeding, microbial aerosols have gradually become the main factor of environmental pollution in animal houses. They not only lead to a large-scale outbreak of infectious diseases, but they also have a certain impact on the health of animals and employees in the houses and increase the difficulty of prevention and control of animal-borne diseases. This paper reviews the distribution, harm, and control measures of microbial aerosols in animal house environments in order to improve people's understanding of them.

## Introduction

Stable colloidal systems formed by microorganisms suspended in the air with dry solid particles and liquid particles are called microbial aerosols (1, 2), which are an important indicator of ambient air quality. Since 1900, researchers have increasingly focused on microbial aerosols. Studies have found that microbial aerosols have posed a great threat to human and livestock health. They not only cause air pollution, but also make animals and humans sick.

Recently, the epidemic of Corona Virus Disease 2019 (COVID-19) has been spreading all over the world. Studies have shown that the main transmission methods of the novel coronavirus are respiratory droplets and close contact. In addition, it is possible to become infected by the novel coronavirus by long-term exposure to aerosols in a relatively closed environment (3). The World Health Organization (WHO) guidelines for the prevention of COVID-19 point out that some medical care processes in the diagnosis and treatment of patients can also lead to a risk of novel coronavirus spreading through aerosols (4, 5). This has led us to think about the risk of environmental exposure to aerosols.

Aerosol transmission happens in not only our daily environment, but also in the closed environments of livestock and poultry houses. As a large breeding country, intensive breeding methods have widespread popularity in China. The concentration of microbial aerosols rise inside and outside animal houses due to the high density of animal breeding, the relatively small space, the physiological characteristics and living habits of poultry, and other reasons (6, 7). Studies have confirmed that high concentrations of microbial aerosols can reduce the resistance of animals and cause serious harm to their production performance and health (8–11).

In the past 20 years, the avian influenza virus has been seriously harming the development of the poultry industry. In 2014, the highly pathogenic avian influenza H5N6 subtype broke out in China and Vietnam. Researchers found through this epidemic event that the virus can also infect humans (12). Porcine reproductive and respiratory syndrome virus (PRRSV), porcine epidemic diarrhea virus (PEDV), and African swine fever virus (ASFV) are the most harmful pathogens in production. It is reported that they can all be transmitted by virus aerosols (13–16).

**Figure 1:**
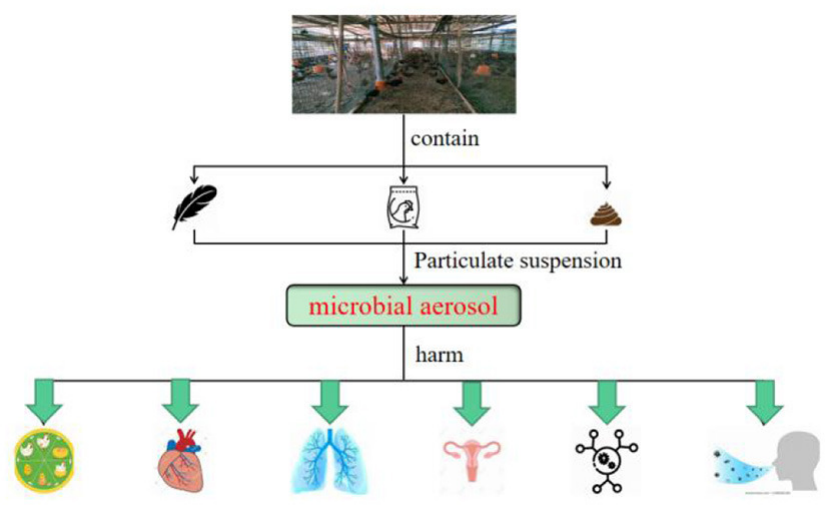
Hazards of microbial aerosol in animal house.

A very small amount of pathogenic microorganisms in the air is enough to cause human and animal diseases. Even if inhaling a high concentration of non-pathogenic microorganism aerosol, the immunity load of animals or human body is increased and body resistance is decreased (17–19). Thus, microbial aerosols cannot be ignored. Therefore, this paper mainly summarizes the distribution characteristics, hazards, transmission, occupational exposure effects, and prevention and control measures of environmental microbial aerosols in animal houses. One of the goals is to attract the attention of relevant personnel (see Figure 1).

## Types and distribution of microbial aerosols

Microbial aerosol which is a colloidal system contains various microbial components, such as bacteria, viruses, Mycoplasma, Chlamydia, Rickettsia, exosomes, etc., it is formed by microorganisms existed widely in nature. They were found to play a vital role in the pathogen transmission of respiratory diseases (20–23). According to the different main components of microbial aerosols, they can be divided into bacterial aerosols, fungal aerosols, and virus aerosols (24), of which bacterial and fungal aerosols account for a relatively large proportion (25). For many years, the monitoring of microbial aerosol concentrations and distributions in indoors environment and atmospheric environment has always been an important topic for scholars.

There were significant differences in bacterial aerosols in different seasons in Beijing's outdoor environment. Bacterial aerosols that can enter the lower respiratory tract in winter (≤ 4.7 μm) accounted for the highest proportion at 61% (26). Similarly, Lanzhou, an important industrial base in China, had the following distribution characteristics of outdoor atmospheric microbial aerosol. The particle size distribution of bacteria, fungi, and *Actinomycetes* in different environmental functional areas in Lanzhou had seasonal characteristics. The particle size of atmospheric fungal aerosol was mainly 0.65–4.7 μm. Grade VI (0.65–1.1 μm) mold aerosol in the air of railway stations and provincial hospitals accounted for more than 35% (27). As an important component of air pollution, these microbial aerosols have negative effects on health, which must be given attention by the public.

Closed indoor environments are more suitable for microbial growth and reproduction. About 85% of human activities are carried out indoors (28). Therefore, it is of importance to study the characteristics of indoor microbial aerosol. For example, a library is a learning place that college students often visit. Zhang found that there is fungal aerosol pollution in different functional divisions of a university library in Xi'an, which was mainly concentrated in grade IV (2.1–3.3 μm) and V (1.1–2.1 μm), accounting for 33.4 and 25.5% (29). Bacterial aerosol pollution existed in different places of a campus in Beijing, including gymnasiums, classrooms, canteens, and other places, and the average concentration of indoor bacterial aerosol was significantly higher than that outdoors. In addition, the study also found that the distribution was 1.1–4.7 μm, accounting for about 70%, of which 1.1–3.3 μm bacterial aerosol accounted for nearly 50% (30). These microbial aerosols with small particle size can penetrate into the deep part of the respiratory tract and bronchioles, seriously endangering human health. In summary, there is a problem of microbial aerosol pollution in the human daily environment.

The environmental composition of animal houses is complex. The species and relative abundance of microbial aerosols are different in different animal houses. In-depth analysis of the types and concentrations of microbial aerosols in animal houses can be used to better evaluate their air quality. For example, Liu identified 13 genera and 25 species of fungi in fungal aerosol from a chicken house in Hebei Province. Its dominant genera were *Aspergillus* and *Penicillium*, and their relative abundance was higher than 50% (31). The dominant bacterial genera of bacterial aerosol in different chicken houses were *Faecalibacterium, Streptomyces, Micromonospora*. In addition, compared with pig houses and cattle houses, the concentration of microbial aerosol in chicken houses is relatively high (32). At present, most of the monitoring in chicken house environments is for the quantitative and qualitative analysis of bacterial aerosols, but there is relatively little research on fungal aerosols. In poultry production, bacterial aerosols and fungal aerosols are important sources of pathogens. Comprehensive analysis can help us better understand the environmental conditions in chicken houses and facilitate the formulation of a more comprehensive disease-prevention and control plans.

According to some reports, potential pathogens in pig houses include *Acinetobacter, Staphylococcus, Pseudomonas, Staphylococcus aureus*, and *Enterococcus avium* (7, 33). The dominant genera of airborne fungi in pig houses are *Dothideomycetes* and *Sordariomycetes* (34). Li found that *Enterbacter* and *Pseudomonas* are the dominant genera of aerobic Gram-negative bacteria after analyzing the airborne microorganisms in a pig house (35). Complete intensive feeding pattern (CP) has become dominant in pig production systems (36). Pigs housed in intensive systems live in much smaller space. The aim is to reach slaughter-age early through limiting their activity and feeding the animals a high-protein diet. With the popularization of this pattern, the environment situation in pig houses is very important to the health of pigs. Therefore, actively monitoring and analyzing microbial aerosol in pig houses will help to enrich the basic data of the health situation in pig houses and give important guidance for future health assessment.

Milk is an important source of nutrients and energy for human beings, so the environments of cowsheds have also received much attention. Duan et al. collected microbial aerosols in six cattle houses in Shandong Province (37). After calculation and analysis, it was found that there were high concentrations of microbial aerosols in cattle houses, and most of them were small particles. Pavan et al. conducted a qualitative analysis on airborne fungi inside and outside a cowshed (38). The results showed that *Cladosporium sp., Aspergillus sp.*, and *Alternaria alternata* were relatively abundant fungal species. Bacterial contamination could also be seen in the air of Czech cattle houses, and *Staphylococcus* and *Streptococcus* were the most identified Gram-positive bacteria (39). There are many literature reviews of the effects of environmental microorganisms in dairy farms in Europe. According to Quintana, the existence of lactic acid bacteria in the air would influence the qualities of the milk (40). Specially in summer, a greater content of spores existed in feces. These spores can contaminate the dairy farm environment (41). Therefore, microbial aerosols in cattle houses are not only the cause of animals' sickness, but are also closely related to the production of raw milk.

The characteristics of fungal and bacterial aerosols in some animal houses are described above. Virus aerosols are also an important component, especially for some zoonosis such as avian influenza, Japanese encephalitis virus, foot and mouth disease virus. The pathogenicity of avian influenza virus to animals and humans is related closely with the role of aerosols. Aerosol exposure increased the likelihood of chicken infection with low pathogenic avian influenza (42). H9N2 AIV from chicken houses were able to infect guinea pigs by aerosol transmission (43). Japanese encephalitis virus (JEV) can cause fatal or serious consequences to both humans and pigs. JEV from swine is considered to be transmitted among mice through aerosols (44). These pathogens affects the growth of swine. Cattle are the primary host of Influenza D Virus (IDV) and the main susceptible animal of foot and mouth disease virus (FMDV) (45, 46). IDV *via* aerosol to a seronegative calf is occurred under experimental conditions (45). FMDV Asia 1 strain was transmitted from pigs to cattle through aerosol under experimental conditions (46). It can be seen that virus aerosols play an important role in the transmission of zoonosis.

More dominant bacteria genera in animal house environments are presented in Table 1. The monitoring and analysis of microbial aerosols in animal houses could provide basic data for controlling the ambient air quality of livestock and poultry houses and provide a theoretical basis and technical support for the healthy breeding and safe production of livestock and poultry. The information suggests that people should pay more attention to the negative effects of microorganisms in the houses and the health of workers.

**Table 1:** Dominant bacteria genus in different animal house.

**Animal house**	**Dominant bacteria genus**	**Ref**.
Pig house	*Enterobacter, Pseudomonas*	Li et al. (35)
Chicken house	*Faecalibacterium, Streptomyces, Micromonospora*	Zhang (32)
Rabbit house	*Enterobacteriaceae*	Duan (47)
Mink house	*Pasteurella, Pseudomonas*	Zhong (48)
Cow barn	*Staphylococcus, Bacillus*	Liu (49)

## Hazards of microbial aerosol

Exposure to microbial aerosols has a negative impact on health because microbial aerosols can invade the body through skin damage, mucosa, the respiratory tract, and the digestive tract. They can then cause irreparable damage to various systems of the body (47, 50). Airborne aerobic bacteria smaller than 2.0 μm can enter the body through respiration. Some of them deposit in the bronchi and bronchioles, affecting the gas exchange in the lungs. Others enter the circulatory system with the exchange of gas and blood, thus causing more serious harm to human and animal health (17). Bertrand found that low-concentration endotoxin exposure increases lung inflammation (51), and microbial aerosol exposure aggravates chronic obstructive pulmonary disease (52).

The size of a particle determines how deeply it enters the respiratory tract, and aerodynamic particles with size <2.5 μm are defined as PM_2.5_ (53). A large number of studies have confirmed that short-term or long-term exposure to environmental PM_2.5_ has extensive damaging effects on human health (54), including reduced lung function, pneumonia, and pulmonary fibrosis. For example, exposure to PM_2.5_ in Ningxia's atmospheric environment was related to lung function. Long-term exposure to a high concentration of PM_2.5_ reduces lung function (55). With increased exposure to PM_2.5_, FVC and FEV1 decreased. In Canada, short-term exposure to environmental PM_2.5_ was associated with the risk of hospitalization and death (56).

In addition, it is widely accepted that PM_2.5_ induces lung inflammation through oxidative stress, thereby causing lung damage (57). More researchers have paid attention to the distribution and inflammatory effect of PM_2.5_ in animal houses. Li analyzed PM_2.5_ collected from poultry farms and found that it contained a large number of potential pathogenic bacteria, such as *P. aeruginosa* (58). The synergy of PM_2.5_ and *P. aeruginosa* caused serious pathological damage to the lungs of mice and aggravates the inflammatory response.

Component analysis of PM_2.5_ in animal houses can be found in many studies, but there are few articles that combine PM_2.5_ in animal houses with lung injury in workers or animals. Correlation analysis between the two could help to find the best disease-prevention strategy and achieve healthy breeding. Of course, we should not only focus on the lungs, but also pay attention to the negative effects on other organs and on performance.

In the production of livestock and poultry, the impact of microbial aerosols on the production performance of livestock and poultry must also be considered. Animal production performance is an important factor that affects economic benefits. Studies have shown that microbial aerosols have a negative impact on animal production performance. For example, Chen found that the weight of mice was significantly lower than that of a control group after 7 days of nasal drip of environmental particles collected from a pigsty, and the weight showed a trend of first decreasing and then increasing during the test (59). Yu formed a microbial aerosol environment with different concentrations by adopting different cleaning methods liking ventilation time and frequency of troughs cleaning sterilization and bedding replacement (9–11). The concentration of microbial aerosols showed an increasing trend with the deterioration of sanitary conditions in the duck house. A high concentration of microbial aerosol in a duck house significantly reduced the average daily gain (ADG) of meat ducks from 54 g/d in 4 fourth week to 21 g/d in eighth week.

In intensive breeding, the growth performance of animals determines the economic benefits. Therefore, the loss caused by microbial aerosols should be reduced as much as possible. Research on the harm of microbial aerosols mostly focuses on the organic damage of the respiratory system and cardiovascular system. Supplementing production-related data such as body weight, average daily gain, and feed meat ratio is helpful to analyze the health hazards of microbial aerosols. There are few reports on the impact of microbial aerosols in animal houses on the health of animals and employees. More studies have begun to explore the distribution characteristics of microbial aerosols in livestock and poultry houses, but there is still a long way to go in research on the harm of microbial aerosols to animals.

## Effect of microbial aerosol propagation

The wide distribution and spread of microbial aerosols lead to environmental pollution, the spread of some infectious diseases, and health threats to nearby residents and animals. The transmission routes of infectious diseases mainly include air transmission, contact transmission, and droplet transmission (60). However, with the deepening of researchers' understanding of particles, the limitations of contact transmission and droplet transmission were gradually being exposed. The researchers could not well explain the view that workers more than three feet apart are at risk of infection. Transmission of undefined small particle aerosols caused respiratory and gastrointestinal infections of viruses. In order to solve this limitation, Jones (60) proposed the concept of aerosol transmission about infectious disease. In recent years, aerosol propagation has been mentioned many times and has been a great concern for researchers.

Animals growing in animal houses can be the first to be threatened by aerosol transmission. Marek's virus (MDV) and African swine fever virus (ASFV) are typical examples. MDV can fall off into the environment with feathers. Respiratory diseases possibly occur if chickens inhale contaminated dust or feather follicle dander (61–63). Marek's disease (MD) results in the formation of lymphomatous lesions in nerves and visceral organs (64). Farms infected with ASFV showed up to 100% mortality 7 days after clinical symptoms (65). If the infected swine cannot be eliminated in time, it is a hidden danger to the swine of the same pig house (66).

*Staphylococcus* and *Erysipelas* were detected in the microbial aerosol of pig house by researchers (67). *Staphylococcus* is considered to be the main cause of skin disease in pigs (68). *Erysipelothrix* is associated with acute septicaemia and endocarditis and arthritis (69). Avian pathogenic *Escherichia coli* (APEC) remains one of the major endemic diseases afflicting the poultry industry worldwide. It causes *airsacculitis*, septicemia and other mainly extraintestinal diseases in chickens. The difference of infection pathway affects the pathological characteristics of APEC. Aerosol infection is considered to be the most serious route of APEC lesions (70). Therefore, the spread risk of microbial aerosol in the house cannot be underestimated.

Microbial aerosols can spread to surrounding areas and pose potential risks to residents' health (71). Li et al. found that microbial aerosols produced by garbage and sewage treatment stations diffuse to the surrounding environment under the action of wind (72). Furthermore, with the extension of diffusion distance, children living in the downwind direction are more vulnerable than other young people (72). Not only can microbial aerosols in animal houses spread to the surrounding environment of animal houses, they can also be detected thousands of meters downwind of animal houses. This causes a wider range of health risk effects and increases the difficulty of prevention and control of animal-borne diseases.

Cowling found that aerosol transmission is one of the important transmission modes of influenza A virus (73). Song also found that microbial aerosol is an important transmission route of antibiotic resistance genes in pig houses (74). It can be seen that aerosol transmission not only can be pathogenic to the animals raised in animal houses (7) but can also pose a threat to the health of farm employees and surrounding residents. Aerosol transmission increases the difficulty of disease prevention and control. In the process of livestock and poultry breeding, aerosol transmission must be fully considered. Minimizing the negative impact caused by aerosol transmission could help ensure the health of workers and surrounding residents.

## Effect of occupational exposure on respiratory system of employees

The composition of microbial aerosols is not stable and is easily affected by other factors. In a specific occupational environment, microorganisms can be transported by air flow, forming a high-concentration microbial aerosol environment. The occurrence of some human diseases is also related to the composition of particulate matter in different places, such as garbage collection sites (75, 76) and breeding farms (77, 78).

According to Muzaini, workers working in sewage treatment plants face various health risks, among which lung-related diseases are one of the main health effects (79). Early studies on occupational exposure risk in a breeding environment showed that compared with ordinary farmers, farmers involved in poultry and pig breeding had a higher probability of suffering from respiratory diseases (80, 81). Due to the increase of microbial aerosol concentration in chicken houses, workers' chances of contacting pathogens increase, and their probability of developing respiratory diseases such as asthma and obstructive pulmonary disease is also increased (82).

Mbareche found *Moraxela spp*. aerosol in a pig house (83). It is also a dominant bacterium in the nasopharyngeal flora of feeding workers (84). The bacteria of this genus are opportunistic pathogens causing upper and lower respiratory tract infections (85), and workers are at risk. Bacteria and fungi account for the vast majority of microbial aerosols, so their impact on health should not be underestimated.

However, a large number of livestock workers are at risk due to respiratory transmission of zoonotic diseases, particularly bovine tuberculosis and brucellosis (86). As early as 1953, Oltramare proposed that brucellosis is an occupational disease of butchers (87). Males working in slaughterhouses had the highest seropositivity rate. This was inseparable from their working time and the lack of professional knowledge about zoonotic nature, such as consuming raw meat and directly contacting the blood and tissues of infected animals (88). Moreover, human-animal contacts at cattle markets and slaughterhouses are also acted as the risk factor for tuberculosis transmission (89). The study demonstrated that the prevalence of latent tuberculosis infection (LTBI) and pulmonary tuberculosis among livestock workers were high in Mexico, it was robustly related to occupational exposure (90).

Globally, outbreaks of Avian Influenza Virus (AIV) continue to burden economies and endanger human, poultry and mammal health. Close contact with infected birds is considered to be the main risk factor for avian influenza infection (91–93). Serological studies are widely used to detect the positive rate of avian influenza virus. It has been proved that a high positive detection rate was seen in the serum of workers involved in live poultry sales and breeding (94). The HPAIV H5N1 showed strong zoonotic characteristics and it was transmitted from birds to mammal including humans (95). Therefore, employees should pay attention to personal protection when engaging in production activities to reduce the risk of infection.

Endotoxin is also a major risk factor in occupational exposure. Endotoxin is a component in the cell walls of Gram-negative bacteria that is released after cell lysis. It is also a common pollutant (96). Total mixed rations and silage were collected from a dairy house in Lithuania and analyzed, and the content of endotoxin was the highest in mixed rations (97). The level of endotoxin in a hen house in Egypt was also high, with an average concentration of 2.23 × 10^5^ CFU/m^3^ (98). It can be seen that endotoxin pollution is common in livestock and poultry houses (99–102).

Endotoxin exposure has various negative impacts on health. For example, there is a correlation between endotoxin exposure and lung disease. As early as 1986, Brigham and Meyrick proposed that endotoxin has a significant effect on the structure and function of the intact lungs of animals and may even have a pathogenic link (103). A Polish study found that exposure to low concentrations of endotoxin is associated with a decrease in forced expiratory volume in 1 s (FEV1) (104). High concentrations of endotoxin exposure were also associated with an increase in hospital visits for asthma (105). Therefore, in some specific workplaces, such as livestock and poultry farms, landfills, etc., workers should be aware of the risks and use appropriate personal protective equipment during their daily work to reduce the incidence of lung diseases.

The presence and concentration of microbial aerosols in the occupational environment are significantly related to the health of employees, and working in an environment with high microbial aerosol concentration for a long time causes many adverse health effects to employees. The impact of occupational exposure on human health may be far more serious than we thought. With the rapid development of the breeding industry, various occupational exposure studies could urge researchers to find effective prevention and control measures, which could provide new ideas for improving the working environment of employees.

## Prevention and control measures for microbial aerosol

The danger of aerosols depends on pathogens, and some pathogens can cause serious infectious diseases. At this point, it is particularly important to prevent and control infectious diseases. Control the source of infection, cut off the route of transmission, and protect vulnerable people. These three steps are routine methods for preventing and controlling infectious diseases. The most important way of all is eliminating to the source of infection, and ensuring that animals are not infected by pathogens, especially zoonotic infections. When engaging in breeding and production, the infected animals with obvious clinical symptoms are found, and the infected animals are isolated in time. If it is found to be a classI animal epidemic, it must be reported immediately, and the diseased animals or even the dead animals must be treated harmlessly, such as chemical preparation or burial.

How to cut off the transmission route is as follows. At present, the following three methods are used to reduce the concentration of microbial aerosol. The first is keeping the livestock and poultry houses clean. The second method is strengthening ventilation. Ventilation has been proven to significantly reduce (but not completely eliminate) the amount of microbial aerosols in the air (18, 106). Reasonable ventilation in a livestock and poultry house provides fresh air and removes dust particles, pathogenic microorganisms, moisture, and harmful gases in the air. Furthermore, it maintains the appropriate temperature and humidity to help control of the small environment (107, 108). In addition, a kind of Nanofiber with high capture capacity has also been found, which may have potential applications for efficient capture of aerosols and viruses (109, 110). The last method is using disinfectants reasonably. In the process of disinfection, it should be ensured that every corner of the house is cleansed of microorganisms that may remain as much as possible to achieve environmental disinfection and reduce the possibility of microbial aerosol transmission.

Lastly, in the process of breeding production, employees can reduce aerosol concentration through standardized workflow. At the same time, the health of employees can be protected through vaccine prevention.

## Perspectives

The air quality in livestock and poultry houses is closely related to the survival and growth of animals. As an important indicator of air quality, microbial aerosols have a far-reaching impact on the health of animals and employees, so it is very important to study them. In the future, we hope that more people will pay attention to the environmental conditions in animal houses and provide a theoretical basis for the healthy breeding of animal. In addition, in the process of site selection and construction of livestock and poultry houses, factors of aerosol transmission, climate, and wind direction should be considered comprehensively in our opinions.

Future research also should continue to monitor the composition and concentration of microbial aerosols in different farms to fill the gaps in related fields. Studies are also being done on equipment or adsorbents that trap or reduce microbial aerosol particles, which can reduce the concentration of aerosols and reduce the risk of disease in animals and humans.

Reducing the occupational risk of microbial aerosol exposure is also an important research topic. The research on microbial aerosols is of great significance to animal health breeding. Such studies could accelerate the standardization and scientific breeding of livestock and poultry breeding industry, as well as lay a theoretical foundation for limiting exposure risk and the prevention and control of microbial aerosols in livestock and poultry houses.

## Conclusion

Microbial aerosols have great significance in the assessment of the environmental status of animal houses. We have mainly summarized the hazards of these aerosols and the risks of transmission and occupational exposure. The results provide a theoretical basis for further study on the damage mechanism of such systems.

## Author contributions

Conceptualization: BW. Writing original daft preparation: CL and YB. Preparation of manuscript: TC, GH, TL, and YG. Editing and revised: CL, YB, and BW. Supervision and funding acquisition: HY and BW. All authors have read and agreed to the published version of the manuscript.

## Funding

This work was supported by the National Natural Science Foundation of China Youth Science Foundation (32102752), the Guangdong Provincial Key Laboratory of Animal Molecular Design and Precise Breeding (2019B030301010), and the Key Laboratory of Animal Molecular Design and Precise Breeding of Guangdong Higher Education Institutes (2019KSYS011).

## Conflict of interest

The authors declare that the research was conducted in the absence of any commercial or financial relationships that could be construed as a potential conflict of interest.

## Publisher's note

All claims expressed in this article are solely those of the authors and do not necessarily represent those of their affiliated organizations, or those of the publisher, the editors and the reviewers. Any product that may be evaluated in this article, or claim that may be made by its manufacturer, is not guaranteed or endorsed by the publisher.

## References

[1] Che FX Li JS Chai TJ. Introduction to Aerobiology. Principles and Applications of Aerobiology. Beijing: Science Press (2004). p. 134–7.

[2] Che FX Yu XH. Theory and Technology Application of Air Microbial Sampling. Beijing: Encyclopedia of China Publishing House (1998). p. 15.

[3] General Office of National Health Commission of the People's Republic of China; Office of National Administration of Traditional Chinese Medicine. COVID-19 diagnosis and treatment protocol (Trial Version 7). Chin Med. (2020) 15:801–5. 10.3760/j.issn.1673-4777.2020.06.001

[4] World Health Organization. Clinical Management of COVID-19: interim guidance. 2020?.05.27. Available online at: https://apps.who.int/iris/handle/10665/332196

[5] World Health Organization?. Infection prevention and control during health care when coronavirus disease (COVID-19) is suspected or confirmed. 2020.06.29. Available online at: who.int

[6] Zhong ZB Chai TJ Duan HY Miao ZM Yao ML Yuan W. Identification of airborne *Staphylococcus aureus* isolated from chicken houses and their spreading around the farms. Acta Veterinaria et Zootechnica Sinica. (2008) 10:1395–401. 10.3321/j.issn:0366-6964.2008.10.016

[7] Hong PY Li X Yang X Shinkai T Zhang Y Wang X Mackie RI. Monitoring airborne biotic contaminants in the indoor environment of pig and poultry confinement buildings. Environ Microbiol. (2012) 14:1420−31. 10.1111/j.1462-2920.2012.02726.x 22414212

[8] Banhazia TM Seedorf J Laffrique M Rutleya DL. Identification of the risk factors for high airborne particle concentrations in broiler buildings using statistical modelling. Biosyst Eng. (2008) 101:100–10. 10.1016/j.biosystemseng.2008.06.007

[9] Yu GL Liu JY Wang Y Cai YM Chai TJ Gao J. Effects of microbial aerosols on stress and production performance of ducks in winter duck culture mode. J Anim Physiol An N. (2015) 27:3402–10. 10.3969/j.issn.1006-267x.2015.11.011

[10] Yu GL Wang Y Wang SG Duan CM Wei LM Gao J. Effects of microbial aerosol in poultry house on meat ducks' immune function. Front Microbiol. (2016) 7:1245. 10.3389/fmicb.2016.01245 27582731 PMC4988117

[11] Yu GL Wei LM Liu YY Liu JY Wang Y Gao J. Influence of indoor microbial aerosol on the welfare of meat ducks. Brit Poultry Sci. (2016) 57:12–22. 10.1080/00071668.2015.1122739 26594822

[12] Wang XJ. Pathogenicity of Avian Influenza Virus H6N6 Subtype of Chicken in Mice and Replication in Swine and Human Respiratory Tissues (Master's Thesis). Guangxi Medicine University, Nanning, China (2019).

[13] Zhao D Liu R Zhang X Li F Wang J Zhang J. Replication and virulence in pigs of the first African swine fever virus isolated in China. Emerg Microbes Infect. (2019) 8:438–47. 10.1080/22221751.2019.1590128 30898043 PMC6455124

[14] Li X Tian K. African swine fever in China. Vet Rec. (2018) 183:300–1. 10.1136/vr.k3774 30194128

[15] Gavier-Widen D Stahl K Dixon L. No hasty solutions for African swine fever. Science. (2020) 367:622–4. 10.1126/science.aaz8590 32029612

[16] Liu Y Zhang X Qi W Yang Y Liu Z An T. Prevention and control strategies of African swine fever and progress on pig farm repopulation in China. Viruses. (2021) 13:2552. 10.3390/v13122552 34960821 PMC8704102

[17] Douwes J Thorne P Pearce N Heederik D. Bioaerosol health effects and exposure assessment: progress and prospects. Ann Occup Hyg. (2003) 47:187–200. 10.1093/annhyg/meg032 12639832

[18] Fiegel J Clarke R Edwarde DA. Airborne infectious disease and the suppression of pulmonary bioaerosols. Drug Discov. (2006) 11:51–7. 10.1016/S1359-6446(05)03687-1 16478691 PMC7108402

[19] Zhang HS Qin M Chai TJ Miao ZM Huang R Liu DJ. Effects of different breeding environments on broilers' immune function. Journal of Huazhong Agricultural University. (2011) 30:34–8. 10.13300/j.cnki.hnlkxb.2011.01.020

[20] Ashiq S Huassain M Ahmad B. Natural occurrence of mycotoxins in medicinal plants: a review. Fungal Genet and Biol. (2014) 66:1–10. 10.1016/j.fgb.2014.02.005 24594211

[21] Hu XQ Wu CY Zhang F Ding WJ. Role of exosomes in respiratory diseases. J Environ Occup Med. (2022) 39:561–9. 10.11836/JEOM21364

[22] Lee H Zhang D Zhu Z Dela CCS Jin Y. Epithelial cell-derived microvesicles activate macrophages and promote inflammation *via* microvesiclecontaining microRNAs. Sci Rep. (2016) 6:35250. 10.1038/srep35250 27731391 PMC5059671

[23] Yu F Jun A Saburo I Kenji K Nobuyoshi K Yusuke Y. Suppression of autophagy by extracellular vesicles promotes myofibroblast differentiation in COPD pathogenesis. J Extracell Vesicles. (2015) 4:28388. 10.3402/jev.v4.28388 26563733 PMC4643181

[24] Shu Y Wang SP Wu LY Zeng Y Zhou Y Hu XX. Review of pathogenic microbial aerosol detection and prevention on livestock farms. Hunan Agric Sci. (2014) 19:36–38, 42. 10.16498/j.cnki.hnnykx.2014.19.001

[25] Lee G Yoo K. A review of the emergence of antibiotic resistance in bioaerosols and its monitoring methods. Rev Environ Sci Biotechnol. (2022) 21:799–827. 10.1007/s11157-022-09622-3 35694630 PMC9169023

[26] Luo YY Mao YX Zhuang SQ Deng FC Hou M Tang S. Seasonal distribution characteristics of bacterial aerosols and their correlations with environmental factors in Beijing, China. Res Environ Sci. (2022) 35:556–65. 10.13198/j.issn.1001-6929.2021.12.02

[27] Chen E Chu KC Luo T Zhao XY Wang RT Liu X. Study on the particle size and distribution characteristics of atmospheric microbial aerosol in different seasons in Lanzhou. Environmental Monitoring in China. (2022) 38:85–95. 10.19316/j.issn.1002-6002.2022.02.11

[28] Zapata-Morín P Reyna-Martinez R Orue N Treviño-Rangel RdeJ Elizondo-Zertuche M Adame-Rodríguez J. The influence of carpeting, human activity and number of beds on airborne fungi concentration in hotel bedrooms. Appl Sci. (2021) 11:6773. 10.3390/app11156773

[29] Zhang Y Kong QQ Wu DM Li AG Gao R. Characteristics of fungal aerosol concentration and particle size and distribution in a university bibrary in Xi'an. J Xi'an Univ of Arch & Tech. (2020) 52:296–301+308. 10.15986/j.1006-7930.2020.02.020

[30] Zhang JP Yin HQ Wang Z Li AG Cao GQ. Study on the indoor microbial aerosol concentration level in campus buildings in the winter, Beijing. Build Sci. (2020) 36:156–65. 10.13614/j.cnki.11-1962/tu.2020.06.21

[31] Liu YW Zhang X Bai FJ Shi YX Wang B Liu N. Dynamic study on the species and concentration of fungi in aerosol of henhouse. Chin J Vet Sci. (2017) 37:2095–100. 10.16303/j.cnki.1005-4545.2017.11.11

[32] Zhang XQ. Microbial community characteristics and antibiotic resistance genes (ARGs) of microbial aerosol in different livestock houses (Master's Thesis). Jilin Agricultural University, Changchun, China (2018).

[33] Tang Q Huang K Liu J Shen D Dai P Li Y. Seasonal variations of microbial assemblage in fine particulate matter from a nursery pig house. Sci Total Environ. (2020) 708:134921. 10.1016/j.scitotenv.2019.134921 31771854

[34] Kumari P Woo C Yamamoto N Choi HL. Variations in abundance, diversity and community composition of airborne fungi in swine houses across seasons. Sci Rep. (2016) 6:37929. 10.1038/srep37929 27892507 PMC5124938

[35] Li C Hao HY Sun LY Chai TJ Wang HR. Airborne microbiological of swine houses monitoring. Acta Veterinaria et Zootechnica Sinica. (2014) 45:1684–92.

[36] Li XJ Wang M Xue Y Duan D Li C Ye J. Characterization and comparison of the bacterial community between complete intensive and extensive feeding patterns in pigs. AMB Express. (2021) 11:32. 10.1186/s13568-021-01191-y 33630191 PMC7907295

[37] Duan HY Zhu YH Liang Y. Detection of microbiological aerosol concentration in cow houses. China Herbivore Science. (2013) 33:47–51.

[38] Pavan R Manjunath K. Qualitative analysis of indoor and outdoor airborne fungi in cowshed. J Mycol. (2014) 2014:8. 10.1155/2014/985921

[39] Matković K Vučemilo M Vinković B Šeol B Pavičić Ž Matković S. Qualitative structure of airborne bacteria and fungi in dairy barn and nearby environment. Czech J Anim Sci. (2007) 8:249–53. 10.17221/2280-CJAS

[40] Quintana ÁR Seseña S Garzón A Arias R. Factors affecting levels of airborne bacteria in dairy farms: a review. Animals. (2020) 10:526. 10.3390/ani10030526 32245161 PMC7142656

[41] Calamari L Morera P Bani P Minuti A Basiricò L Vitali A. Effect of hot season on blood parameters, fecal fermentative parameters, and occurrence of Clostridium tyrobutyricum spores in feces of lactating dairy cows. J Dairy Sci. (2018) 101:4437–47. 10.3168/jds.2017-13693 29501337

[42] Jegede A Fu QG Lin M Kumar A Guan JW. Aerosol exposure enhanced infection of low pathogenic avian influenza viruses in chickens. Transbound Emerg Dis. (2018) 66:435–44. 10.1111/tbed.13039 30307712

[43] Lv J Wei BZ Yang Y Yao ML Cai YM Gao YW. Experimental transmission in guinea pigs of H9N2 avian influenza viruses from indoor air of chicken houses. Virus Res. (2012) 170:102–8. 10.1016/j.virusres.2012.09.003 23022529

[44] Chai CX Palinski R Xu YX Wang Q CaoSJ Geng Y. Aerosol and contact transmission following intranasal infection of mice with Japanese encephalitis virus. Viruses. (2019) 11:87. 10.3390/v11010087 30669601 PMC6356382

[45] Salem E Hägglund S Cassard H Corre T Näslund K Foret C. Pathogenesis, host innate immune response, and aerosol transmission of influenza D virus in cattle. J Virol. (2019) 93:e01853–18. 10.1128/JVI.01853-18 30674628 PMC6430558

[46] Colenutt C Gonzales JL Paton DJ Gloster J Nelson N Sanders C. Aerosol transmission of foot-and-mouth disease virus Asia-1 under experimental conditions. Vet Microbiol. (2016) 189:39–45. 10.1016/j.vetmic.2016.04.024 27259825

[47] Duan HY Wang L Chai TJ. Detection of airborne microbiological aerosol in rabbit stables. China Herbivore Science. (2005) 25:41–4.

[48] Zhong ZB. Detection of bacterial aerosol and endotoxin in mink breeding houses. China Animal Health Inspection. (2015) 32:22–26, 32. 10.3969/j.issn.1005-944X.2015.11.006

[49] Liu Y. Airborne Microorganism Flora Composition of Cow Barn and Its Effect on Milk (Master's Thesis). Northeast Agricultural University, Harbin, China (2016).

[50] Wen ZB Chen Y Du Q Yang WH Li JS Hu LF. Contamination of microbiological aerosol generated by pathogenic microbiological labs. Mil Med Sci. (2013) 37:1–5. 10.7644/j.issn.1674-9960.2013.01.001

[51] Bertrand C Salvador-Cartier C Schmidlin F Eutamene H Bueno L Chovet M. LPS-induced lung inflammation is linked to increased epithelial permeability: role of MLCK. Eur Respir J. (2005) 25:789–96. 10.1183/09031936.05.00064704 15863634

[52] Devries R Kriebe D Sama S. Low level air pollution and exacerbation of existing COPD: a case crossover analysis. Environ Health-glob. (2016) 15:98. 10.1186/s12940-016-0179-z 27756407 PMC5070120

[53] Zhao CX Wang YQ Wang YJ Zhang HL Zhao BQ. Spatial and temporal distribution of PM25 and PM10 pollution levels and their relationship with meteorological conditions in Beijing in winter and spring. Environ Sci. (2014) 35:418–27. 24812928

[54] Karimi A Shirmardi M Hadeie M Birganif YT Neisif A Takdastanf A. Concentrations and health effects of short- and long-term exposure to PM_2.5_, NO_2_, and O_3_ in ambient air of Ahvaz city, Iran (2014–2017). Ecotoxicol Environ Saf. (2019) 180:542–8. 10.1016/j.ecoenv.2019.05.026 31128552

[55] Tian D Chen X Hou P Zhao Y Zhao Y Zhang Y. Effects of exposure to fine particulate matter on the decline of lung function in rural areas in northwestern China. Environ Sci Pollut Res Int. (2022) 29:14903–13. 10.1007/s11356-021-16865-0 34623588

[56] Shin HH Gogna P Maquiling A Parajuli RP Haque L Burr B. Comparison of hospitalization and mortality associated with short-term exposure to ambient ozone and PM25 in Canada. Chemosphere. (2021) 265:128683. 10.1016/j.chemosphere.2020.128683 33158503

[57] Ren H Lu J Ning J Su X Tong Y Chen J. Exposure to fine particulate matter induces self-recovery and susceptibility of oxidative stress and inflammation in rat lungs. Environ Sci Pollut Res Int. (2020) 27:40262–76. 10.1007/s11356-020-10029-2 32661967

[58] Li M Wei XL Li YZ Feng T Jiang LL Zhu HW. PM_2.5_ in poultry houses synergizes with *Pseudomonas aeruginosa* to aggravate lung inflammation in mice through the NF-κB pathway. J Vet Sci. (2020) 21:e46. 10.4142/jvs.2020.21.e46 32476320 PMC7263920

[59] Chen GX Liu M Zhang HF Guo ZD Liu LN Liu JB. Effects of environmental particles in closed fattening pig house on lung injury in mice. Chin J Anim Dis. (2018) 54:112–6.

[60] Jones RM Brosseau LM. Aerosol transmission of infectious disease. J Occup and Environ Med. (2015) 57:501–8. 10.1097/JOM.0000000000000448 25816216

[61] Biggs PM Nair V. The long view: 40 years of Marek's disease research and avian pathology. Avian Pathol. (2012) 41:3–9. 10.1080/03079457.2011.646238 22845316

[62] Couteaudier M Denesvre C. Marek's disease virus and skin interactions. Vet Res. (2014) 45:36. 10.1186/1297-9716-45-36 24694064 PMC4030002

[63] Bavananthasivam J Alqazlan N Alizadeh M MatsuyamaKato A Astill J Kulkarni RR. The regulatory microenvironment in feathers of chickens infected with very virulent Marek's disease virus. Viruses. (2022) 14:112. 10.3390/v14010112 35062316 PMC8781056

[64] Baigent S Davison F. Marek's disease virus: biology and life cycle. Marek's Disease: An Evolving Problem (Biology of Animal Infections). Compton, UK: Academic Press (2004). p. 62–77. 10.1016/B978-012088379-0/50010-4

[65] Guberti V Khomenko S Masiulis M Kerba S. African Swine Fever in Wild Boar Ecology and Biosecurity. Rome, Italy: FAO, OIE and EC (2019). p. 8.

[66] Brellou GD Tassis PD Apostolopoulou EP Fortomaris PD Leontides LS Papadopoulos GA. Report on the first African swine fever case in Greece. Vet Sci. (2021) 8:163. 10.3390/vetsci8080163 34437485 PMC8402752

[67] Cui H Zhang C Liu J Dong S Zhao K Chen L. The distribution characteristics of aerosol bacteria in different types of pig houses. Animals. (2022) 12:1540. 10.3390/ani12121540 35739876 PMC9219456

[68] Foster AP. *Staphylococcal* skin disease in livestock. Vet Dermatol. (2012) 23:342–51. 10.1111/j.1365-3164.2012.01093.x 22823580

[69] Ebwanga EJ Ghogomu SM Paeshuyse J. Molecular characterization of ASFV and differential diagnosis of erysipelothrix in ASFV-infected pigs in pig production regions in cameroon. Vet Sci. (2022) 9:440. 10.3390/vetsci9080440 36006355 PMC9416451

[70] Paudel S Fink D Abdelhamid MK Zöggeler A Liebhart D Hess M. Aerosol is the optimal route of respiratory tract infection to induce pathological lesions of colibacillosis by a lux-tagged avian pathogenic *Escherichia coli* in chickens. Avian Pathol. (2021) 50:417–26. 10.1080/03079457.2021.1978392 34505551

[71] Kassander A Richard J. A study of the trajectories and diffusion patterns of ground-generated airborne particulates under orographic wind-flow conditions. J Atmos Sci. (2010) 16:617–25.

[72] Li P Li L Yang K Zheng TL Liu JX Wang YJ. Characteristics of microbial aerosol particles dispersed downwind from rural sanitation facilities: size distribution, source tracking and exposure risk. Environl Res. (2021) 195:110798. 10.1016/j.envres.2021.110798 33529647

[73] Cowling BJ Ip DK Fang VJ Suntarattiwong P Olsen SJ Levy J. Aerosol transmission is an important mode of influenza A virus spread. Nat Commun. (2013) 4:1935. 10.1038/ncomms2922 23736803 PMC3682679

[74] Song L Wang C Jiang GY Ma JB Li YF Chen H. Bioaerosol is an important transmission route of antibiotic resistance genes in pig farms. Environ Int. (2021) 154:06559. 10.1016/j.envint.2021.106559 33864959

[75] Madsen AM Frederiksen MW Jacobsen MH Tendal K. Towards a risk evaluation of workers' exposure to handborne and airborne microbial species as exemplified with waste collection workers. Environ Res. (2020) 183:109177. 10.1016/j.envres.2020.109177 32006769

[76] Madsen AM Alwan T Orberg A Uhrbrand K Jørgensen MB. Waste workers' exposure to airborne fungal and bacterial species in the truck cab and during waste collection. Ann Occup Hyg. (2016) 60:651–68. 10.1093/annhyg/mew021 27098185 PMC4915520

[77] Li ZX. Study on the Temporal and spatial distribution microbial aerosols, particulate matter and harmful gas in enclosed layer house (Master's Thesis). Southwest University of Science and Technology, Mianyang, China (2021).

[78] Qiao TT Guo Y Liu JQ Bai YY Yu H Duan JW. Veterinary food hygiene branch of Chinese society of animal husbandry and veterinary medicine. Study on the difference of the fungal aerosol of sheep house with different structures and different ways of feeding. Jinan, China (2019). p. 62–3.

[79] Muzaini K Yasin SM Ismail Z Ishak AR. Systematic review of potential occupational respiratory hazards exposure among sewage workers. Front Public Health. (2021) 9:646790. 10.3389/fpubh.2021.646790 33763402 PMC7982603

[80] Samoli E Stafoggia M Rodopoulou S Ostro B Alessandrini E Basagaña X. Which specific causes of death are associated with short term exposure to fine and coarse particles in Southern Europe? Results from the MED-PARTICLES project. Environ Int. (2014) 67:54–61. 10.1016/j.envint.2014.02.013 24657768

[81] Zhao QJ Liu XJ Zeng XL Bao HR. Effect of PM_2.5_ on the level of nuclear factor erythroid-2 related factor 2 in chronic obstructive pulmonary disease mice and its relationship with oxidative stress. Zhonghua Yi Xue Za Zhi. (2016) 96:2241–5. 27480657 10.3760/cma.j.issn.0376-2491.2016.28.009

[82] Viegas S Faísca VM Dias H Clérigo A Carolino E Viegas C. Occupational exposure to poultry dust and effects on the respiratory system in workers. J Toxicol Env Heal. (2013) 76:230–9. 10.1080/15287394.2013.757199 23514065

[83] Mbareche H Veillette M Pilote J Létourneau V Duchaine C. Bioaerosols play a major role in the nasopharyngeal microbiota content in agricultural environment. Int J Env Res and Pub He. (2019) 16:1375. 10.3390/ijerph16081375 30995814 PMC6518280

[84] Cormier Y Tremblay G Meriaux A Brochu G Lavoie J. Airborne microbial contents in two types of swine confinement buildings in Quebec. Am Ind Hyg Assoc J. (2010) 51:304–9. 10.1080/15298669091369709 2353639

[85] Laura PVM Kristian R. Virulence mechanisms of *Moraxella* in the pathogenesis of infection. Curr Opin Infect Dis. (2009) 22:279–85. 10.1097/QCO.0b013e3283298e4e 19405217

[86] Mukthar MM Mahamudul H Sadia PF. Occupational exposure to livestock and risk of tuberculosis and brucellosis: a systematic review and meta-analysis. One Health. (2022) 15:e100432. 10.1016/j.onehlt.2022.100432 36277098 PMC9582573

[87] Oltramare M Despres P. Brucellosis, an occupational disease of butchers. Praxis. (1953) 42:678–84. 13100226

[88] Aworh MK Okolocha E Kwaga J Fasina F Lazarus D Suleman I. Human brucellosis: seroprevalence and associated exposure factors among abattoir workers in Abuja, Nigeria-2011. Pan Afr Med J. (2013) 16:103. 10.11604/pamj.2013.16.103.2143 24876892 PMC4033582

[89] Jenkins AO Cadmus SI Venter EH Pourcel C Hauk Y Vergnaud G. Molecular epidemiology of human and animal tuberculosis in Ibadan, Southwestern Nigeria. Vet Microbiol. (2011) 151:139–47. 10.1016/j.vetmic.2011.02.037 21458174

[90] Torres-Gonzalez P Soberanis-Ramos O Martinez-Gamboa A Chavez-Mazari B Barrios-Herrera MT Torres-Rojas M. Prevalence of latent and active tuberculosis among dairy farm workers exposed to cattle infected by *Mycobacterium bovis*. PLoS Negl Trop Dis. (2013) 7:e2177. 10.1371/journal.pntd.0002177 23638198 PMC3636137

[91] Mostafa A Abdelwhab EM Mettenleiter TC Pleschka S. Zoonotic potential of influenza A viruses: a comprehensive overview. Viruses. (2018) 10:497. 10.3390/v10090497 30217093 PMC6165440

[92] Van Kerkhove MD Mumford E Mounts AW Bresee J Ly S Bridges CB. Highly pathogenic avian influenza (H5N1) pathways of exposure at the animal-human interface, a systematic review. PLoS ONE. (2011) 6:e14582. 10.1371/journal.pone.0014582 21283678 PMC3025925

[93] De Marco MA Delogu M Facchini M Di Trani L Boni A Cotti C. Serologic evidence of occupational exposure to avian influenza viruses at the wildfowl/ poultry/ human interface. Microorganisms. (2021) 9:2153. 10.3390/microorganisms9102153 34683475 PMC8539340

[94] Li X Tian B Jianfang Z Yongkun C Xiaodan L Wenfei Z. A comprehensive retrospective study of the seroprevalence of H9N2 avian influenza viruses in occupationally exposed populations in China. PLoS ONE. (2017) 12:e0178328. 10.1371/journal.pone.0178328 28575073 PMC5456037

[95] Kalthoff D Globig A Beer M. (Highly pathogenic) avian influenza as a zoonotic agent. Vet Microbiol. (2010) 140:237–45. 10.1016/j.vetmic.2009.08.022 19782482

[96] Han C Yang T Han YP Jiang YG Xiao BY Liu QX. Research status of endotoxin pollution in bioaerosols of wastewater treatment plants. Journal of Tianjin Chengjian University. (2022) 28:37–42.

[97] Vaičiuliene G Bakutis B Jovaišiene J Falkauskas R Gerulis G Kerziene S. Prevalence of mycotoxins and endotoxins in total mixed rations and different types of ensiled forages for dairy cows in lithuania. Toxins. (2021) 13:890. 10.3390/toxins13120890 34941727 PMC8707214

[98] Ahmed MFE Ramadan H Seinige D Kehrenberg C Abd El-Wahab A Volkmann N. Occurrence of extended-spectrum beta-lactamase-producing *Enterobacteriaceae*, microbial loads, and endotoxin levels in dust from laying hen houses in Egypt. BMC Vet Res. (2020) 16:301. 10.1186/s12917-020-02510-4 32838780 PMC7446189

[99] Schulze A van Strien R Ehrenstein V Schierl R Küchenhoff H Radon K. Ambient endotoxin level in an area with intensive livestock production. Ann Agric Environ Med. (2006) 13:87–91. 16841878

[100] Thorne PS Ansley AC Perry SS. Concentrations of bioaerosols, odors, and hydrogen sulfide inside and downwind from two types of swine livestock operations. J Occup Environ Hyg. (2009) 6:211–20. 10.1080/15459620902729184 19177273 PMC4844821

[101] Ko G Simmons OD 3rd Likirdopulos CA Worley-Davis L Williams M Sobsey MD. Investigation of bioaerosols released from swine farms using conventional and alternative waste treatment and management technologies. Environ Sci Technol. (2008) 42:8849–57. 10.1021/es801091t 19192808

[102] Gladding TL Rolph CA Gwyther CL Kinnersley R Walsh K Tyrrel S. Concentration and composition of bioaerosol emissions from intensive farms: pig and poultry livestock. J Environ Manage. (2020) 272:111052. 10.1016/j.jenvman.2020.111052 32669254

[103] Brigham KL Meyrick B. Endotoxin and lung injury. Am Rev Respir Dis. (1986) 133:913–27. 3085564

[104] Cyprowski M Sobala W Buczyńska A Szadkowska-Stańczyk I. Endotoxin exposure and changes in short-term pulmonary function among sewage workers. Int J Occup Med Env. (2015) 28:803–11. 10.13075/ijomeh.1896.00460 26224492

[105] Mendy A Wilkerson J Salo PM Weir CH Feinstein L Zeldin DC. Synergistic association of house endotoxin exposure and ambient air pollution with asthma outcomes. Am J Respir Crit Care Med. (2019) 200:712–20. 10.1164/rccm.201809-1733OC 30965018 PMC6775869

[106] Bartlett KH. Evaluation and determinants of airborne bacterial concentrations in school classrooms. J Occup Environ Hyg. (2004) 1:639–47. 10.1080/15459620490497744 15631055

[107] Yang ZF Zhong XQ Yu J Yang HM. Environmental regulation of white feather broiler feeding and management: a review. Jiangsu Agricultural Sciences. (2020) 48:53–6.

[108] Ruzal M Shinder D Malka I Yahav S. Ventilation plays an important role in hens' egg production at high ambient temperature. Poultry Sci. (2011) 90:856–62. 10.3382/ps.2010-00993 21406372

[109] Alali H Ai Y Pan YL Videen G Wang C. A collection of molecular fingerprints of single aerosol particles in air for potential identification and detection using optical trapping-raman spectroscopy. Molecules. (2022) 27:5966. 10.3390/molecules27185966 36144702 PMC9505655

[110] Shen H Zhou Z Wang H Chen J Zhang M Han M. Photosensitized electrospun nanofibrous filters for capturing and killing airborne coronaviruses under visible light irradiation. Environ Sci Technol. (2022) 56:4295–304. 10.1021/acs.est.2c00885 35262328

